# First episode psychosis Post-COVID 19 infection : case report

**DOI:** 10.1192/j.eurpsy.2023.1712

**Published:** 2023-07-19

**Authors:** S. Dhakouani, R. Kammoun, N. Hamrouni, M. Karoui, F. Ellouze

**Affiliations:** psychiatry, Razi hospital, tunis, Tunisia

## Abstract

**Introduction:**

During the course of COVID-19 pandemic, the respiratory system is the most commonly affected while many neuropsychiatric manifestations of the disease have been observed.

**Objectives:**

Emphasize the importance of detecting psychiatric symptoms in patients infected with Covid 19.

**Methods:**

Presentation of case report

**Results:**

A 44-year-old woman with no personal history of chronic diseases and with no psychiatric family history. About her experience with covid 19, her mother died as a result of covid 19 infection and our patient had been hospitalized for 17 days for pulmonary infection covid 19, during her hospitalization in COVID 19 unit she presented insomnia and anxiety without behavioural disorders. She had a good evolution of the respiratory symptoms and she was discharged under corticotherapy and anticoagulants.

She was admitted in our department after 45 days of her covid 19 infection for acute behavioural disorders.

On physical examination: she was hemodynamically stable and well oriented. Neurological examination was with no abnormalities. Cerebral CT scan was normal and lumbar puncture was indicated and the analysis of the CSF did not reveal any anomalies. At the psychiatric interview she was extremely agitated, anxious and hallucinated, she had disorganised speech with derailment and neologisms, she was disinhibited and her mood was exalted. She presented also a delusion of grandeur and delusion of persecution.

**Conclusions:**

In individuals presenting with COVID-19 infection, consideration should be made for psychiatric manifestations because COVID-19 diagnosis predispose vulnerable patients to psychosis.

**Disclosure of Interest:**

None Declared

